# The threshold effect of factors associated with spontaneous abortion in human-assisted reproductive technology

**DOI:** 10.1038/s41598-021-90970-5

**Published:** 2021-05-31

**Authors:** Fei Li, AiQin Niu, XingMei Feng, Ying Yan, Ying Chen

**Affiliations:** 1 Department of Center for Reproductive Medicine, The First People’s Hospital of Shangqiu, Henan, People’s Republic of China; 2 Department of Molecular Biology, The First People’s Hospital of Shangqiu, Henan, People’s Republic of China

**Keywords:** Diseases, Reproductive disorders, Infertility

## Abstract

We explored the independent risk factors associated with cases of spontaneous abortion in infertile patients treated with human-assisted reproductive technology (ART) and established a smooth curve fit and perform a threshold effect analysis can provide guidance and a valuable reference for predicting the probability of spontaneous abortion. This was a retrospective cohort study of 16,097 patients successfully conceived with ART in Shangqiu First People's Hospital from June 2013 to December 2018. Overall, 2,378 (14.77%) had an abortion and 13,719 (85.23%) did not have an abortion. Multivariate logistic regression analysis showed that female age (OR 1.050; 95% CI 1.032–1.069; *P* < 0.001), male age (OR 1.100; 95% CI 1.086–1.115; *P* < 0.001), follicular-stimulating hormone (OR 1.049; 95% CI 1.022–1.076; *P* < 0.001), anti-Mullerian hormone (OR 0.893; 95% CI 0.862–0.925; *P* < 0.001) and the number of fetuses at pregnancy diagnosis were independent factors associated with spontaneous abortion. The threshold effect analysis found that when female age > 32 years (cut-off point) old, age and the risk of spontaneous abortion were positively correlated. When follicular-stimulating hormone > 6.1 IU/L (cut-off point), follicular-stimulating hormone was positively correlated with the occurrence of spontaneous abortion, When anti-Mullerian hormone ≤ 3.1 ng/mL (cut-off point), anti-Mullerian hormone was negatively correlated with the occurrence of spontaneous abortion and there was a linear positive correlation between antral Follicle Counting and live birth. In addition, the older the male age, the higher the incidence of abortion. The smooth curve fit and threshold effect analyses can provide a more detailed estimate of the probability of spontaneous abortion for pregnant couples.

## Introduction

Assisted reproductive technology (ART) is a common choice for infertility patients to achieve pregnancy, and various embryo manipulation techniques and controlled ovarian stimulation specifications increasingly improve^1^. However, technological progress does not prevent and avoid spontaneous abortion (SAB), and a study showed that the incidence of early pregnancy loss in ART patients receiving fresh embryo transplantation is 14.9%^2^. Although many studies have been performed to identify predictors of spontaneous abortion, there is still no real consensus, which may be because the underlying pathogenesis and influencing factors of spontaneous abortion have not been fully clear^3,4^. Considering the burden of spontaneous abortion on families and society, it is necessary to explore and determine the prevalence and risk factors linked to miscarriage in infertile couples, especially for infertile couples assisted by ART. The independent risk factors associated with cases of spontaneous abortion in infertile patients can provide guidance and a valuable reference for predicting the probability of spontaneous abortion.

To date, spontaneous abortion has become one of the most common complications of human pregnancy in ART treatment^5^. It has become a thorny issue that endangers the safety of pregnant women and fetuses, and many factors contribute to miscarriage. Hahn KA et al. showed that female age affects oocyte and endometrial quality in ART, thus causing abortion^3^. Other studies have shown that assisted fertility treatment itself may lead to decreased embryo quality and endometrial receptivity changes, which are risk factors for abortion, and these problems greatly reduce the pregnancy success rate of ART^6,7^. Research by Daiet et al. showed that the probability of spontaneous abortion in women ≥ 40 years old was significantly higher than that in women under 40 years old^8^. A study conducted by Iwayoma et al. on Japanese men also showed that an increase in male age was closely related to the high incidence of spontaneous abortion, with statistical significance^9^. However, given that aging is a complex process, it is difficult to define the threshold point to make it clear that all events have the same result. In addition, ART patients are usually older and more likely to have some reproductive endocrine and pelvic diseases as well as immune system abnormalities^10,11^. Patients receiving ART-assisted pregnancy also require large doses of ovarian stimulation drugs, which further increase the possibility of fetal growth restriction and abortion^6^. According to previous studies, and considering the emotional and psychological burden spontaneous abortion imposes on infertile couples offered ART, awareness of the risk factors associated with spontaneous abortion is of significant help. Through the smooth points curve and threshold effect analysis, we can provide a more detailed estimate of the probability of spontaneous abortion for these patients.

Previously, the clinical management of spontaneous abortion in ART patients was primarily based on small studies, and the relationship between male age and the risk of spontaneous abortion in patients receiving ART is scarce^6,8^. Therefore, a large and comprehensive study on the risk of abortion in ART patients is necessary. Our study retrospectively analyzed the clinical data of 16,097 patients who received ART treatment and successfully conceived at the Shangqiu First People's Hospital from June 2013 to December 2018. The individual parameters and laboratory data of both husbands and wives were included to explore the associated factors of spontaneous abortion. Through the independent associated factors, we can draw a smooth curve fit based on these factors and analyze the threshold effect so that we can more accurately reveal the correlation between miscarriage and independent associated factors. Through individual parameters and laboratory test results, we can predict the probability of miscarriage to carry out preventive and targeted interventions to prevent miscarriage and inform patients in advance of the risk of miscarriage to avoid patients having too low of an awareness of unpredictable pregnancy risks, resulting in anxiety and conflicts between doctors and patients. This study aimed to determine the prevalence and risk factors linked to spontaneous abortion in infertile couples treated with ART to provide a reference and help for clinical work.

At our reproductive medical center, > 10,000 IVF/ICSI cycles are performed each year, and this could be the first study to establish a smooth curve fit and threshold effect analysis that systematically evaluates the independent risk factors associated with cases of spontaneous abortion in infertile patients treated with human-assisted reproductive technology, and we discuss possible underlying explanatory mechanisms, with the goal of providing guidance for predicting the probability of spontaneous abortion of such patients in future clinical practice.

## Material and methods

### Ethical approval and informed consent

This study strictly followed the relevant requirements of the Declaration of Helsinki of the World Medical Association. The study was a retrospective cohort approved by the Medical Ethics Committee of Shangqiu First People's Hospital, and the requirement for informed consent was waived by the Medical Ethics Committee of Shangqiu First People's Hospital. All methods were performed in accordance with the relevant guidelines and regulations.

### Subjects

We analyzed clinical data from 16,097 patients who successfully conceived using ART treatment at the Shangqiu First People's Hospital from June 2013 to December 2018. Patients who underwent ART treatment used their own oocytes, and the embryos transferred were fresh embryos. The experimental subjects were divided into an "abortion group" and a "non-abortion group" according to the outcome of abortion. Among them, there were 2,378 cases in the abortion group and 13,719 cases in the non-abortion group. Patient characteristics, such as female age, male age and female cause of infertility, were self-reported. Other indicators, such as body mass index (BMI), follicular-stimulating hormone (FSH), estradiol (E2), luteinizing hormone (LH), anti-mullerian hormone (AMH), controlled ovulation induction protocol, etc., were obtained by a professional medical technician. Data were compiled from the hospital electronic medical record system. Each patient was identified by their unique medical record number, and our data did not include identifiable participant data for the purpose of safeguarding patient privacy. All of the included case data were recorded and sorted by dedicated personnel. The inclusion criteria included patients who met the study criteria, patients who received ART treatment using their own oocytes and the embryos transferred were fresh-embryo, complete laboratory data, and women who did not receive preimplantation genetic testing (PGT). Beforehand, we formulated the following strict criteria for the rejection of individuals: a history of recurrent miscarriage and uterine fibroids, endocrine and metabolic diseases; severe uterine malformations; chromosomal abnormalities in both spouses, combined with severe diabetes, thyroid hyperfunction and other diseases; untreated hydrosalpinx; stage III or IV endometriosis; untreated endometrial disease; drug allergies; follow-up loss; and other factors.

### Diagnostic criteria for spontaneous abortion

The spontaneous abortion diagnosis comes after the pregnancy is confirmed, and the clinical history, physical examination, female endocrine and vaginal ultrasound confirm that the embryo stops developing before 20 weeks of pregnancy^12^.

### Influencing factors associated with spontaneous abortion

We compared the influencing factors of patients in the spontaneous abortion group and the non-abortion group, these factors that were choosen for analysis were female age, male age, BMI, FSH, LH, E2, P, AMH, prolactin (PRL), number of treatment cycles, number of transferred embryos, method of fertilization, embryo stage, number of fetuses and controlled ovulation induction protocol, and we determined the statistically significant associated factors between the two groups of patients. Based on these results, further single-factor and multi-factor logistic regression analyses were performed to screen independent associated factors that were associated with spontaneous abortion in ART. Then, a smooth fitting curve model was constructed based on the independent associated factors, and the linearity between these associated factors and the occurrence of SAB was observed. This relationship is further analyzed based on the model observation results, and finally, the risk of SAB is explored in detail based on the results before and after the threshold inflection point of each independent associated factor.

### Treatment selection for controlled ovulation induction

There patients were put on appropriate controlled ovarian induction protocols by experienced physicians according to the patient’s baseline characteristics, followed by embryo transfer in the same cycle. The starting dose of gonadotropin(Puregon, Organon, The Netherlands) were administrated on the basis of patient`s antral follicle count, age, BMI and previous ovarian response to stimulation. The dosage was also adjusted constantly according to patient`s response. When dominant follicles measuring > 16 mm account for 60% or a follicle reached 20 mm in mean diameter, trigger was normally performed using 250 ug r-hCG (Livzon Pharmaceuticals, China) in combination with 2000 IU u-HCG(Merck Schlano, Italy). Oocyte retrieval under the guidance of transvaginal ultrasound was performed 37 h after trigger.

### Statistical analysis

All analyses were performed using the statistical package R (The R Foundation; http://www.r-project.org; version 3.6.1), EmpowerStats (http://www.empowerstats.com) and SPSS 19.0 (IBM, Armonk, NY, USA) software. Continuous variable data are expressed as the mean ± standard deviation (Mean ± SD) and were compared using the Student’s t-test or Wilcoxon rank-sum test; categorical variables are expressed as frequencies (percentages) and were compared using the chi-square test. We then applied multiple regression analysis to estimate the independent relationship between spontaneous abortion and female age, male age, basal FSH, AMH, the number of treatment cycles and the number of fetuses at pregnancy diagnosis, with an adjustment for potential confounders. *P* < 0.05 indicates that the difference is statistically significant.

For independent associated factors (male age, female age, FSH, AMH, the number of treatment cycles and the number of fetuses at pregnancy diagnosis), the R 3.6.1 software package was used to further apply a two-piecewise linear regression model to examine the threshold effect of the associated factors on spontaneous abortion using a smoothing function. The threshold level was determined using trial and error. We also conducted a log likelihood ratio test comparing the one-line linear regression model with a two-piecewise linear model and calculated the before and after odds ratios (ORs) and found 95% for independent associated factors threshold breakpoints, with a confidence interval (CIs) of *P* < 0.05 indicating that the difference is statistically significant.

## Results

We collected effective experimental data from 16,097 patients who successfully conceived with ART. In addition, 2378 (14.77%) had miscarriages, and 13,719 (85.23%) did not have a miscarriage. There were statistically significant differences in female age, and the female age of the abortion group was significantly higher than that of the non-abortion group (*P* < 0.001). We also found that the male age of the abortion group was significantly higher than that of the non-abortion group (*P* < 0.001). Body mass index and basal FSH in the abortion group were also significantly higher than those in the non-abortion group; however, basal P, anti-Mulller hormones and prolactin in the abortion group were also significantly lower than those in the non-abortion group. In addition, there was also a statistical difference between the number of treatment cycles, number of transferred embryos and number of fetuses at pregnancy diagnosis between the two groups (Table 1).

**Table 1:** Comparison of baseline data between spontaneous abortion group and non-abortion group.

Projects	Abortion group (n = 2378)	Non-abortion group (n = 13,719)	*t/x*^2^ value	*P* value
Female age (years)	32.63 ± 5.38	30.16 ± 4.40	− 24.377	< 0.001
Male age (years)	38.46 ± 8.95	34.00 ± 9.13	− 22.024	< 0.001
BMI (Kg/M2)	23.12 ± 3.17	22.68 ± 3.35	− 5.853	< 0.001
Basal FSH (IU/L)	7.21 ± 3.08	6.89 ± 2.39	− 5.688	< 0.001
Basal LH (IU/L)	5.61 ± 4.69	5.65 ± 4.44	0.380	0.704
Basal E2 (ng/L)	56.01 ± 148.05	53.33 ± 138.28	− 0.858	0.391
Basal P (μg/L)	0.46 ± 0.37	0.51 ± 0.56	3.339	0.001
AMH (ng/mL)	3.32 ± 2.85	3.67 ± 2.86	3.903	< 0.001
PRL (ng/L)	19.30 ± 27.73	22.55 ± 121.63	1.281	0.200
**Number of treatment cycles (n)**				
1–2	93.1 (2214/2378)	95.9 (13,163/13,719)	40.673	< 0.001
3–4	6.0 (143/2378)	3.7 (504/13,719)		
> 4	0.9 (21/2378)	0.4 (52/13,719)		
**Number of transferred embryos (n)**				
1	18.8 (448/2378)	16.5 (2270/13,719)	9.150	0.010
2	77.9 (1852/2378)	80.5 (11,048/13,719)		
3	3.3 (78/2378)	3.0 (396/13,719)		
**Method of fertilization**				
IVF	53.2 (1740/2378)	71.8 (9856/13,719)	0.001	0.981
ICSI	26.8 (638/2378)	28.2 (3863/13,719)		
**Embryo stage**				
D3	87.6 (2083/2378)	86.7 (11,896/13,719)	1.382	0.251
D5, D6	12.4 (295/2378)	13.3 (1823/13,719)		
**Number of fetuses (n)**				
1	84.7 (2013/2378)	67.3 (9236/13,719)	289.718	< 0.001
2	15.1 (360/2378)	32.2 (4427/13,719)		
3	0.2 (5/2378)	0.4 (60/13,719)		
**Controlled ovulation induction protocol**				
Early-follicular phase GnRH-a long protocol	53.2 (1266/2378)	54.6 (7490/13,719)	1.438	0.487
Mid-luteal phase GnRH-a long protocol	34.3 (816/2378)	33.2 (4566/13,719)		
GnRH antagonist protocol and others	12.5 (296/2378)	12.2 (1673/13,719)		

Data are shown as means ± standard deviation or N(%). BMI, body mass index; FSH, follicular-stimulating hormone; LH, luteinizing hormone; E2, estradiol; P, progesterone; AMH, anti-Mulller hormones; PRL, prolactin; IVF, in vitro fertilization; ICSI, intracytoplasmic sperm injection.

Univariate logistic regression analysis showed female age (OR 1.117; 95% CI 1.107–1.128; *P* < 0.001), male age (OR 1.052; 95% CI 1.047–1.057; *P* < 0.001), body mass index (OR 1.021; 95% CI 1.011–1.032; *P* < 0.001), basic FSH (OR 1.044; 95% CI 1.028–1.061; *P* < 0.001), basic P (OR 0.835; 95% CI 0.751–0.929; *P* = 0.001), anti-Mulller hormones (OR 0.953; 95% CI 0.929–0.976; *P* < 0.001), the number of of treatment cycles (OR 1.254; 95% CI 1.180–1.333; *P* < 0.001) are the predictive factors affecting spontaneous abortion. Multivariate logistic regression analysis showed female age (OR 1.050; 95% CI 1.032–1.069; *P* < 0.001), male age (OR 1.100; 95% CI 1.086–1.115; *P* < 0.001), basic FSH (OR 1.049; 95% CI 1.022–1.076; *P* < 0.001), anti-Mulller hormones (OR 0.893; 95% CI 0.862–0.925; *P* < 0.001) and number of fetuses at pregnancy diagnosis were independent predictors of spontaneous abortion (Table 2).

**Table 2:** Logistic regression analysis of factors related to spontaneous abortion.

Projects	Unadjusted			Adjusted		
	OR	95% C.I	*P* value	OR	95% C.I	*P* value
Female age (years)	1.117	1.107–1.128	< 0.001	1.049	1.032–1.069	< 0.001
Male age (years)	1.052	1.047–1.057	< 0.001	1.100	1.084–1.112	< 0.001
BMI (Kg/M2)	1.021	1.011–1.032	< 0.001	1.016	0.994–1.036	0.133
Basal FSH (IU/L)	1.044	1.028–1.061	< 0.001	1.049	1.022–1.075	< 0.001
Basal P (μg/L)	0.835	0.751–0.929	0.001	1.031	0.954–1.148	0.761
AMH (ng/mL)	0.953	0.929–0.976	< 0.001	0.893	0.861–0.925	< 0.001
PRL (ng/L)	0.998	0.996–1.001	0.200	1.001	0.997–1.003	0.963
Number of treatment cycles	1.254	1.180–1.333	< 0.001	1.100	0.995–1.209	0.052
**Number of transferred embryos (n)**						
1	Reference			Reference		
2	0.923	0.913–0.936	0.005	0.902	0.879–1.042	0.121
3	0.866	0.715–1.028	0.525	0.843	0.707–1.053	0.875
**Number of fetuses(n)**						
1	Reference			Reference		
2	0.536	0.513–0.562	< 0.001	0.326	0.301–0.342	< 0.001
3	0.951	0.915–0.973	< 0.001	0.896	0.853–0.929	< 0.001

Data are shown as means ± standard deviation. BMI, body mass index; FSH, follicular-stimulating hormone; P, progesterone; AMH, anti-Mulller hormones; PRL, prolactin;

Adjusted for female age, male age, body mass index, follicular-stimulating hormone, progesterone, anti-Müllerian hormon, prolactin, number of treatment cycless, number of transferred embryos and number of fetuses except the subgroup variable.

**Figure 1:**
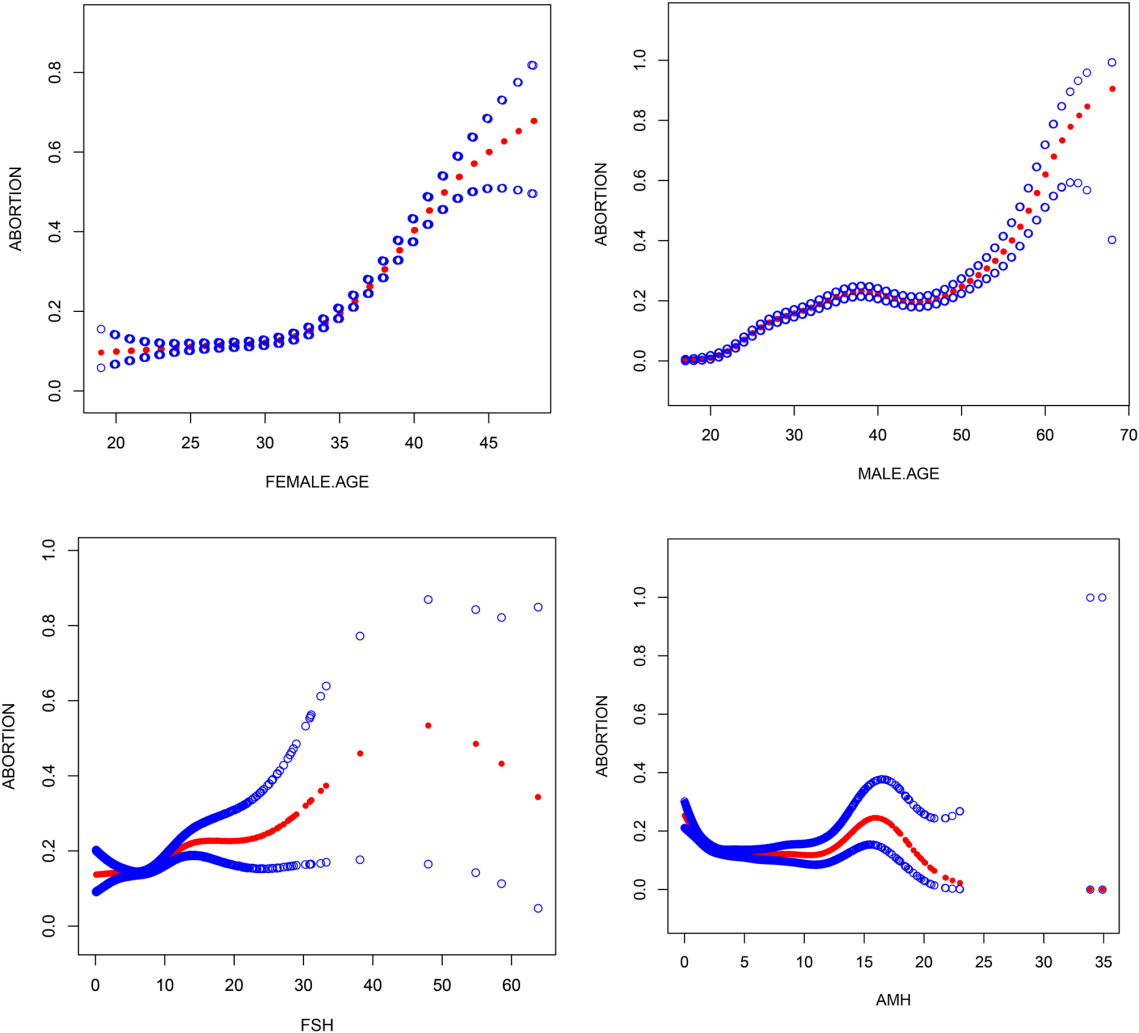
Association between spontaneous abortion and Female age, male age, basal FSH and AMH. A threshold, nonlinear association between spontaneous abortion and these independent predictive factors was found in a generalized additive model(GAM). Solid rad line represents the smooth curve fit between variables. Blue bands represent the 95% of confidence interval from the fit. Pregnancy outcome was defined as live birth rate, Gn, Gonadotropin.

Multivariate logistic regression analysis showed that female age, male age, basic FSH, and anti-Mulller hormones were independent predictors of spontaneous abortion. The smooth curve fit showed that female age, male age, and basic FSH were positively correlated with miscarriage and that AMH had a negative correlation with miscarriage. However, these associated factors do not have a simple linear relationship with the occurrence of SAB (Fig. 1), and further threshold effect analysis is needed.

Through threshold effect analysis, it is found that the female age inflection point is 32 years. When the age is more than 32 years old, the risk of spontaneous miscarriage increases sharply with age (OR 1.2; 95% 1.2–1.2; *P* < 0.001), and when the age is ≤ 32 years, the probability of spontaneous miscarriage and female age are not statistically significant (OR 1.0; 95% 1.0–1.0; *P* = 0.060). The correlation between male age and the probability of spontaneous abortion tends to be more stable; the older the age, the greater the probability of miscarriage (OR 1.1; 95% 1.0–1.1; *P* < 0.001). The threshold point of basic FSH is 6.1 IU/L when FSH > 6.1 IU/L. The incidence of miscarriage increases significantly (OR 1.1; 95% 1.0–1.1; *P* < 0.001) when FSH ≤ 6.1 IU/L. The probability of spontaneous abortion and FSH were not statistically significant (OR 1.0; 95% 0.9–1.0; *P* = 0.603). The threshold points of AMH were 3.1 ng/mL and 11.6 ng/mL. When AMH ≤ 3.1 ng/mL, the incidence of miscarriage is OR 0.8; 95% 0.7–0.8; *P* < 0.001. When 11.6 ng/mL ≥ AMH > 3.1 ng/mL, the incidence of miscarriage is OR 1.0; 95% 1.0–1.0; *P* = 0.365. When AMH > 11.6 ng/mL, the incidence of miscarriage is OR 1.1; 95% 1.0–1.1; *P* = 0.124 (Table 3).

**Table 3:** Threshold effect analysis of spontaneous abortion using piece-wise linear regression.

Projects	Unadjusted	Adjusted
	OR value (95% C.I) *P* value	OR value (95% C.I) *P* value
**Female age (year)**		
≤ 32 years	1.0 (1.0–1.0) 0.056	1.0 (1.0–1.0) 0.060
> 32 years	1.1 (1.0–1.1) < 0.001	1.2 (1.2–1.2) < 0.001
Male age (year)	1.0 (1.0–1.0) < 0.001	1.1 (1.0–1.1) < 0.001
**Basal FSH (IU/L)**		
≤ 6.1 IU/L	1.0 (1.0–1.0) 0.466	1.0 (0.9–1.0) 0.603
> 6.1 IU/L	1.1 (1.0–1.1) < 0.001	1.1 (1.0–1.1) < 0.001
**AMH (ng/mL)**		
≤ 3.1 ng/mL	0.8 (0.6–0.8) < 0.001	0.8 (0.7–0.8) < 0.001
3.1 ng/mL–11.6 ng/mL	1.0 (1.0–1.1) 0.293	1.0 (1.0–1.0) 0.365
> 11.6 ng/mL	1.0 (0.9–1.1) 0.079	1.1 (1.0–1.1) 0.124

FSH, follicular-stimulating hormone; AMH, anti-Mulller hormones;

Adjusted for female age, male age, body mass index, follicular-stimulating hormone, anti-Müllerian hormon, prolactin, number of treatment cycless, number of transferred embryos and number of fetuses except the subgroup variable.

## Discussion

Spontaneous abortion refers to the spontaneous termination of pregnancy before 20 weeks^13^. It is one of the common abnormal pregnancy outcomes, with an incidence of approximately 10–20%^8,14^. It has become a thorny issue that endangers the safety of pregnant women and fetuses. Meanwhile, ART is a relatively effective method for the clinical treatment of infertility. This technology has helped many infertility patients successfully conceive. However, the high risk of spontaneous abortion has again caused huge psychological damage to these patients. Related literature reports and accepts ART. After treatment, the spontaneous abortion rate after pregnancy is approximately 25%^15^, and the early abortion rate before 12 weeks of pregnancy can reach 17.8%^16^. Another study showed that early spontaneous abortion occurs after ART-assisted pregnancy. The probability is approximately 10.6%^17^. In 2017, a large-scale study in China showed that the incidence of early pregnancy loss in ART patients who received fresh embryo transfer was 14.9%^2^. In view of this finding, our study explored the risk factors for miscarriage in patients receiving human-assisted reproductive technology (ART). Through univariate and multivariate logistic regression analysis, it was shown that female age, male age, basic FSH, AMH and the number of fetuses at pregnancy diagnosis influenced spontaneous abortion. For these independent predictors, a smooth curve fit and threshold effect analysis that systematically evaluates the independent risk factors associated with cases of spontaneous abortion was successfully constructed. These research results can be used to assess the risk and probability of spontaneous abortion in patients receiving ART as well as provide preventive and personalized interventions for pregnant couples.

Our study found that the older the female, the higher the risk of miscarriage in ART patients. Through the establishment of a smooth fit curve, we found that female age is an independent predictor of spontaneous abortion and is positively correlated with it, but we found that this is not a simple linear relationship. According to its threshold effect analysis, we found that the inflection point is 32 years old, which means that the probability of spontaneous abortion is relatively stable when the age is ≤ 32 years old in the special population receiving ART-assisted reproductive treatment. However, after age > 32 years, the older the age, the higher the probability of spontaneous abortion. A large number of studies have shown that female age is very important to the quality of embryos and endometrial receptivity. With increasing age, the number and quality of oocytes significantly reduces, the number of mitochondria reduces, there is a significant decrease in ATP content in the cytoplasm, and the proportion of abnormal embryo chromosome structure increases. Spontaneous abortion is closely related to embryo chromosomal abnormalities^18,19^, some studies have shown that chromosomal abnormalities are the most common cause of spontaneous abortion, and 45% to 70% of SABs are caused by chromosomal and genetic abnormalities^20,21^. Another study showed that pregnant women ≥ 35 years of age who had a spontaneous abortion in patients receiving ART was significantly higher than that of women < 35 years of age, and the proportion of autosomal triploid abnormalities was the highest^22^. Research by Daiet et al. showed that the probability of spontaneous abortion in women ≥ 40 years old was significantly higher than that of women under 40 years old^6^. Another study found that more than 50% of spontaneous abortions after ART are closely related to chromosomal abnormalities^23^. In addition, as women age, decreased endometrial receptivity and abnormal blood flow are important causes of SAB. By studying the relationship between female age and miscarriage, we can more accurately predict the probability of miscarriage in ART patients and provide them with scientific guidance and timely intervention to obtain a better pregnancy outcome.

Obviously, female age has been confirmed by numerous studies as an important factor affecting embryonic chromosomal abnormalities and decreased endometrial receptivity, and it directly leads to the occurrence of SAB. However, studies on the relationship between male age and spontaneous abortion risk in ART therapy are insufficient. Our study found that male age is an independent risk factor for spontaneous miscarriage in ART patients, which was positively correlated. Through the smooth fitting curve, we found there was a linear relationship between the items, with an odds ratio of 1.1 (95% CI = 1.0–1.1, *P* < 0.001), suggesting that the probability of abortion increased by 1.1 times with every 1-year increase in male age. A large sample of data from the 2011–2013 and 2013–2015 national surveys in the United States shows that male age is closely related to spontaneous miscarriage. The older the age, the higher the risk of miscarriage^24^. Although the target population was different, this finding was similar to our research results. Another study on patients with natural conception showed that the risk of spontaneous abortion in men over 35 years of age increased by 1.26 times (95% CI = 1.00, 1.60, *P* < 0.001), and it was not related to the presence of alcohol, caffeine, or smoking^25^. A case–control study by Kleinhaus K et al. showed that the odds ratio of spontaneous abortion was 1.6 (95% CI = 1.2, 2.0, *P* = 0.003) when males were ≥ 40 years old and that the risk was independent of female age and other factors^26^. Other studies have shown that the age of men is not only associated with spontaneous abortion, but also increases the risk of autosomal dominant genetic diseases, autism spectrum disorders and schizophrenia. These studies suggest that men over the age of 40 seek genetic counseling before seeking assisted pregnancy treatment^27^. In addition, a study conducted by Iwayoma et al. on Japanese men also showed that the increase in male age was closely related to the high incidence of spontaneous abortion, with statistical significance. Frattarelli et al. also showed that spontaneous abortion was significantly correlated with the increase in the father's age. When the age was > 50, the incidence of spontaneous abortion was 41.5%, and when the age was ≤ 50, the incidence was 24.4%^28^.

The older the male, the higher the reproductive cell mutation rate of the offspring. Various factors associated with increasing age will destroy the DNA replication and repair mechanism and increase the possibility of DNA breakage, thus leading to the occurrence of abortion^29,30^. Although a consensus has been reached on the age of advanced mothers, which is agreed to be 35 years old, there is currently no agreement on the definition of the age of elderly fathers. Given that aging is a complex process, it is difficult to find a clear age critical point because the various risks caused by the age effect will be interfered with by external factors, and it is difficult to define the threshold point to make it clear that all events have the same result. However, research on the possible relationship between male age and the probability of SAB can provide older men with genetic counseling and the prediction of SAB risk probability before pregnancy so that clinicians can formulate preventive treatment measures based on the patient's condition.

This study found that basic FSH and AMH are other important factors leading to spontaneous abortion in ART patients. Both basic FSH and AMH are good indicators for predicting ovarian responsiveness in infertile patients^31,32^. They also play a vital role in assisted reproduction (ART), when ovarian reserve decreases, first, the value of basic FSH increases, while the value of AMH decreases^33^. Ovarian reserve and endometrial conditions are the basis for embryo implantation and growth. Therefore, changes in hormone levels may lead to spontaneous abortion, especially in the ART treatment process, because the use of large doses of follicle stimulating hormone affects the level of female endocrine hormones and the natural selection of eggs. Normally, growing follicles are exposed to higher estrogen levels, so ART treatment itself may affect the quality of embryos and the function of the endometrium, leading to miscarriage^34,35^. Through the establishment of a smooth plot curve, we found that basic FSH was an independent predictor of spontaneous abortion and was positively correlated with it. However, we found that the relationship between these items is not a simple linear relationship. For the analysis of its threshold effect, we found that its threshold turning point is 6.1. When basic FSH ≤ 6.1 IU/L, there was no significant difference in the incidence of miscarriage in the special population treated with ART. However, when FSH > 6.1 IU/L, the odds ratio was 1.1 (95% CI = 1.0–1.1, *P* < 0.001), suggesting that the larger the basic FSH, the higher the probability of spontaneous abortion. Similarly, we found that AMH was also an independent predictor of spontaneous abortion through a smooth fitting curve, but there was still a nonlinear relationship. According to its threshold effect analysis, we found that its threshold breakpoints were 3.1 ng/mL and 11.6 ng/mL. In the special population receiving ART-assisted reproductive treatment, when basic AMH ≤ 3.1 ng/mL, AMH was negatively correlated with the occurrence of miscarriage, and the odds ratio was 0.8 (95% CI = 0.7–0.8, *P* < 0.001), suggesting that the greater the value of AMH, the lower the incidence of spontaneous abortion. However, when AMH > 3.1 ng/mL, according to the analysis of the segmented threshold effect based on the smooth points curve, the probability of spontaneous abortion and AMH was not statistically significant before and after the turning point of 11.6 ng/mL.

In addition, our study showed that the abortion rate of twins/triplets was lower than that of singleton pregnancies, probably because of the different number of successful implantation of embryos between singleton and twin/triplet pregnancies, and the diagnosis of missed abortion was all embryo stops developing before 20 weeks of pregnancy, this means that the pregnancy can continue even if an embryo is lost. A study suggests that the rate of pregnancy loss before 24 weeks of twins is reportedly lower than that of single pregnancies (1.3% in twins versus 4.8% in singletons)^36^, this result was consistent with our research. Moreover, a cohort study of 233 patients (171 in Day 3 cleavage-stage group and 62 in Day 5 blastocyst-stage group) revealed that there were no statistically significant differences with regard to early abortion rate between cleavage-stage group and blastocyst-stage group^37^. Our study also showed that there was no difference in the rate of spontaneous abortion between cleaveds and blastocysts, this suggests that D3 cleavage-stage embryos and D5/D6 blastocyst-stage embryos have the same developmental potential after successful implantation, they differ only in the success rate of implantation of embryos, this result is also consistent with previous researches.

In summary, female age, male age, basic FSH, AMH and the number of fetuses at pregnancy diagnosis are independent associated factors influencing SAB in patients receiving ART. The smooth points curve and threshold effect analysis provide clinical management strategies for these patients. When female age > 32 years, age and the risk of spontaneous abortion were positively correlated; the greater the age, the higher the possibility of a miscarriage. However, when female age ≤ 32 years, age and spontaneous abortion had no obvious statistical significance. When basic FSH > 6.1 IU/L, FSH was positively correlated with the occurrence of spontaneous abortion; the higher the FSH value, the higher the incidence of miscarriage. When AMH ≤ 3.1 ng/mL, AMH was negatively correlated with the occurrence of spontaneous abortion; the higher the AMH value, the lower the incidence of abortion. In addition, there was a linear positive correlation between AFC and live birth; the older the male, the higher the incidence of abortion. In response to these research results, we can predict the probability of SAB based on the individual parameters of infertile patients and the results of laboratory examinations to prevent and target treatments. Our study has some strengths. First, our sample size is relatively large compared with previous and similar studies. Second, we address the nonlinearity in the present study and further explore this factor. Third, we handle target independent variables as both continuous variables and categorical variables. Such an approach can reduce the contingency in the data analysis and enhance the robustness of the results. However, the main limitation of our study is that it is a retrospective cohort, which cannot exclude all potential biases, and women’s pregnancy is a process of continuous change, during which there are many confounding factors that lead to the occurrence of SAB. For example, maternal infection factors, environmental factors, emotional factors, etc., were not taken into account in this study, and prospective, large-scale and multicenter clinical trials are still needed to confirm these factors in the future.
